# Bio-Based Electrospun Fibers for Wound Healing

**DOI:** 10.3390/jfb11030067

**Published:** 2020-09-22

**Authors:** Bahareh Azimi, Homa Maleki, Lorenzo Zavagna, Jose Gustavo De la Ossa, Stefano Linari, Andrea Lazzeri, Serena Danti

**Affiliations:** 1 Interuniversity National Consortium of Materials Science and Technology (INSTM), 50121 Florence, Italy; b.azimi@ing.unipi.it (B.A.); lorenzo@zavagna.it (L.Z.); andrea.lazzeri@unipi.it (A.L.); 2 Department of Civil and Industrial Engineering, University of Pisa, 56126 Pisa, Italy; 3 Department of Carpet, University of Birjand, Birjand 9717434765, Iran; 4 Doctoral School in Life Sciences, University of Siena, 53100 Siena, Italy; josegustavo.delao@student.unisi.it; 5 Linari Engineering s.r.l., 56121 Pisa, Italy; stefano.linari@linarisrl.com

**Keywords:** skin, tissue engineering, biopolymers, biodegradable, nanofiber, wound dressing

## Abstract

Being designated to protect other tissues, skin is the first and largest human body organ to be injured and for this reason, it is accredited with a high capacity for self-repairing. However, in the case of profound lesions or large surface loss, the natural wound healing process may be ineffective or insufficient, leading to detrimental and painful conditions that require repair adjuvants and tissue substitutes. In addition to the conventional wound care options, biodegradable polymers, both synthetic and biologic origin, are gaining increased importance for their high biocompatibility, biodegradation, and bioactive properties, such as antimicrobial, immunomodulatory, cell proliferative, and angiogenic. To create a microenvironment suitable for the healing process, a key property is the ability of a polymer to be spun into submicrometric fibers (e.g., via electrospinning), since they mimic the fibrous extracellular matrix and can support neo- tissue growth. A number of biodegradable polymers used in the biomedical sector comply with the definition of bio-based polymers (known also as biopolymers), which are recently being used in other industrial sectors for reducing the material and energy impact on the environment, as they are derived from renewable biological resources. In this review, after a description of the fundamental concepts of wound healing, with emphasis on advanced wound dressings, the recent developments of bio-based natural and synthetic electrospun structures for efficient wound healing applications are highlighted and discussed. This review aims to improve awareness on the use of bio-based polymers in medical devices.

## 1. Introduction

Skin is an important multifunctional organ of the human body, which acts as a natural barrier against environmental factors and protects the interior tissues from physical, chemical, and biological influences. As the skin performs vital functions, any structural damage, such as large and deep wounds, can be troublesome and requires prompt and effective treatment [1,2,3]. Over the past decades, wound care has progressively become a major worldwide public health concern. Because, inefficient and defective treatment of skin damages can even be fatal. Hence, intensive research has been performed in this area focusing on the development of efficient therapeutic approaches and design of new dressing materials that can improve the wound healing procedure [4,5,6]. For the restoration of the injured tissue, the wound healing process consists of a cascade of events, including hemostasis, inflammation, and proliferation as well as remodeling of the tissue [5,7].

Restoring a protective barrier is the main part of the wound therapy. Depending on the severity and acuteness of the wound, an appropriate dressing with effective protection of the wound site and efficient skin regeneration is clinically essential for accelerating the wound healing [4,8].

Since ancient times, the wound care process has included covering of the injured site using a dressing material that prevents dehydration and infections [9]. In the past decade, wound healing treatments have progressed from traditional treatments using ointments and gauze coverage to advanced multifunctional wound dressings and tissue engineered substitutes. Inexpensive and readily available conventional wound dressings such as gauze and bandages can basically protect the wound from external agents, but they create a dry environment locally, which can lead to complications, such as subsequent infections [2,10]. In recent years, considerable advances have been achieved in designing modern dressings to protect the wound from dehydration and infection, and facilitate the healing process instead of just covering the wound [3,11,12]. Wound dressings in the form of hydrogels [13,14,15], hydrocolloids [16,17,18], sponges [19,20,21,22], alginates [23,24,25], and transparent films [11] have been developed and some of them are commercially available. These materials are different in their inherent features, such as hydrophobicity, permeability, and adsorption capacity.

An ideal wound dressing should isolate the wound from adverse external factors, absorb the exudates from the wound surface, protect the injured site from bacterial infection, have anti- inflammatory function, and induce cell proliferation to facilitate tissue regeneration and boost the healing process. It should provide a suitable moist environment at the wound site, and preserve the tissue from further damage. In the case of non-compressible type of wounds, elasticity is another important requirement for a dressing to avoid wound compression. In addition, the wound dressing material should be soft, biocompatible, non-toxic, and non-allergenic [19,26,27,28]. The effective healing process will improve by applying multifunctional wound dressings. Wound type and its features, healing time, and chemical, physical, and mechanical characteristics of the dressing material should be considered in designing a functional wound dressing [5,7].

Recently, great attention has been placed on fabricating biopolymer-based nanofibrous structures through the electrospinning method. Electrospinning technology is a commonly, low-cost, and tunable procedure for generating ultra-fine fibers with some unique properties. Owing to flexibility in choosing the raw materials and the possibility to tune the ultimate properties, the electrospinning technique has been extensively employed for biomedical materials like tissue engineering scaffolds, wound dressings, and drug delivery systems [7,8]. Electrospun structures composed of fibers with nano-scale diameter are proposed as ideal wound dressings and tissue substitutes due to their similarity to the extracellular matrix (ECM) fibrillar part [11,26,29,30]. Owing to the large specific surface area and high porosity with small pore size, these materials present efficient performance for improving the healing process. Nanofibrous scaffolds with appropriate mechanical properties could be proper substrates for cell proliferation. Besides, the incorporation of therapeutic and pharmaceutical agents is used to functionalize electrospun dressings for efficiently targeting different wound types [12,27,31,32,33,34].

The arrival of new polymers and fabrication methods offers advanced wound dressings with tunable functionalities along with excellent structural and mechanical properties. Bio-based polymeric materials are much proposed for skin regeneration as functional skin substitutes, wound healing patches, and dressings in healing of different types of wounds. A wide range of natural biopolymers (e.g., cellulose, chitosan, gelatin, hyaluronic acid, and collagen) have been used for electrospinning of nanofibers to simulate the native tissue matrix and healing of the wounds. Polylactides (PLAs) and polyhydroxyalkanoates (PHAs) are synthetic bio-based polymers which are commonly applied for electrospinning of wound dressings. The mechanical, degradation, and/or morphological characteristics of wound dressings can be tuned by coaxial, multi-nozzle, or blend electrospinning of natural and bio-based synthetic polymers [35].

**Figure 1:**
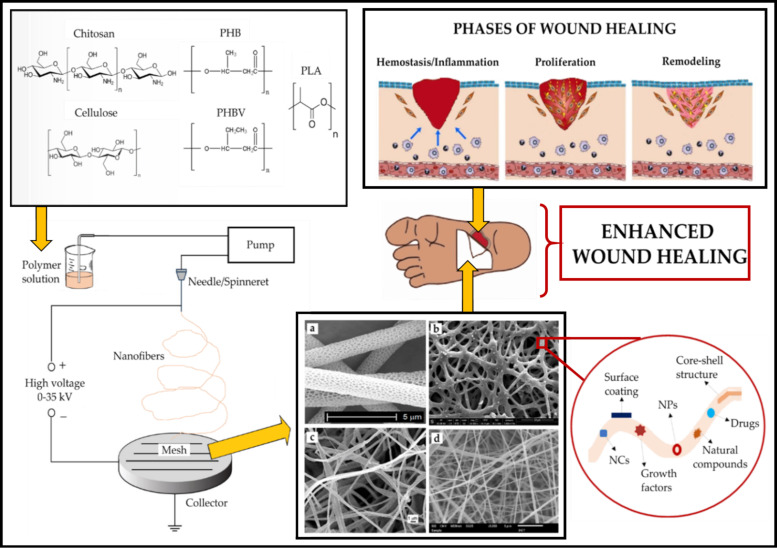
Schematic depicting the topics covered in this review article: biopolymers (natural and synthetics) and their composites processed via electrospinning to produce ultrafine fibers specific for wound healing applications. The schematic of the phases of wound healing reproduced from an open access paper [36] distributed under the terms of the Creative Commons CC BY license, scanning electron microscope (SEM) images (**a**,**b**) are unpublished original pictures by the authors, (**c**) reproduced with permission from [37] (license number: 4873720705310) and (**d**) reproduced with permission from [38] (license number: 4873720243859). The schematic of the foot is adapted with permission from [39] (license numbers: 4875271369979).

Highlighting and explaining the recent progress in electrospinning of different synthetic and natural biopolymers, with a focus on their applications related to wound healing, are the intention of this review (Figure 1). Firstly, the fundamental concepts of wound healing, with emphasis on wound dressings, are presented. Then, the electrospinning method, the advantages of electrospun nanofiber for wound healing applications, and the relationship between the electrospinning variables and efficiency of the designed wound dressing are reported. Thereafter, an overview of recently developed multifunctional bio-polymeric based electrospun structures including cellulose, chitosan, PHAs, and PLA for wound healing are presented and discussed. In the last section, the review encompasses the future prospects of electrospinning biopolymer fibers for wound healing applications.

## 2. Wound Healing

Wound healing is a procedure in which the cells can regenerate and treat the injured tissue [2]. This process occurs across four continuous and partially overlapping phases, consisting of hemostasis, inflammation surrounding the injured region, cellular migration and proliferation, and ECM remodeling (Figure 1) [40,41,42].

The history of wound care dates back to the ancient Sumerian and Egyptian therapies using natural products such as honey, milk, mud, plants, and animal fats. Today, healing approaches have dramatically changed. The wound healing products have evolved over the years from topical ointments to traditional dressings mainly cotton and wool gauzes. Although most of these products provided some benefit to acute wounds they failed to cure chronic and complicated wounds. Therefore, gauze and cotton dressings have been partially replaced with a new generation of dressings. Recently, the main approach relies on preventing, emerging, or eradicating existing infections along with accelerating the healing process with structural and functional regeneration of the skin [20,43].

### Wound Dressing

Numerous research works aiming to enhance the healing of wounds have confirmed the significant benefits and effective role of wound dressings in the healing process. Effectual wound care strongly depends on the appropriate selection of dressings for each specific type of wound [2,44].

A proper wound dressing should have good biocompatibility and mechanical flexibility. Besides, it should enable the maintenance of a moist environment, have permeability, and exudate absorption, provide efficient protection against bacterial infections, and external trauma, and have easy removal without adhesion [43,45].

According to types of injury, wound dressings have been classified in to passive, interactive, advanced, and bioactive. Passive wound dressings such as gauze, absorbent pads, and bandages are known to protect the wound site from bacterial penetration and mechanical trauma. However, they are unable to control the amount of moisture in the wound. These types of dressings are useful for minor wounds. Interactive dressings owing to their flexibility are applicable for wounds in joints and other hard to reach areas of the body. Hydrogel dressings, based on polyurethanes and transparent silicones film or foam are some examples of interactive dressings. Advanced dressings fabricated of alginates, hydrofibers, and hydrocolloids are another type of wound dressing, which facilitates the healing process by controlling the wound environment moisture. Recent research attempts have led to improving the functionality of wound dressings to design bioactive dressings (e.g., skin grafts or substitutes, drug-loaded, and antimicrobial dressings), as the new generation of wound healing products. The resultant dressings effectively enhance wound healing and prolong the usage time of the dressing [2,3,46]. Besides, these multifunctional wound dressings are important to accelerate wound healing through the controlled delivery of therapeutic agents [43,45].

Despite the numerous wound dressings available to date, there is still an essential demand to improve the performance and efficiency of wound dressings. In research of advanced wound therapeutics, electrospun fibers have currently gained great attention as multifunctional wound dressings.

## 3. Electrospinning Process and Its Advantages for Wound Healing Applications

The nanofibrous structures have shown more efficient wound healing compared to traditional dressings, owing to their distinguished features. Among the nanofibers manufacturing methods, electrospinning is a versatile and directly applicable procedure to spin fibrous structures produced from a wide range of materials with controllable features, compositions, shapes, and morphologies [2]. The formation of nanofibers applying the electrospinning technique relies on the uniaxial stretching of the viscoelastic solution or melt under electrostatic forces. Owing to their excellent characteristics, electrospun fibers have the potential to develop textile technology to design advanced biomedical materials in the form of wound dressings, drug carriers, and scaffolds of tissue engineering [47].

The electrospinning process enables the production of interconnected networks from fibers of nano-scale diameter, which are similar to the native structure of the natural ECM, thus promoting the normal functions of the cells, such as supporting attachment and proliferation [48]. Regarding biologically inspired features, large surface area to volume ratio, and the porosity of the electrospun fibrous networks with a small pore size improve the hemostasis at the wound site without the use of a hemostatic agent. Due to the abovementioned physical properties, nanofibers can absorb exudates optimally and provide a moist environment for cell respiration and proliferation. The porous nature of such structures with tiny pores seems to reduce bacterial infection, provides high permeability, and protects the injured tissue from dehydration. The capability and flexibility to incorporate drugs and other bioactive molecules such as growth factors, nanoparticles, antimicrobials, and anti-inflammatory agents, into the nanofibers is another important advantage of the electrospinning process [49]. Electrospun wound dressings can provide compliance and flexibility upon applying and comfortability after placement. In the case of dressings which are fabricated by electrospinning of biodegradable polymers, enhanced patient compliance and comfort may be provided due to less need to change the dressing. The biodegradable electrospun wound dressings also persuade healing and increase cell growth rate due to their high compatibility with blood and tissues. The degradation rate of the scaffolds could be tuned with the rate of tissue regeneration [1,40,46,50,51].

The abovementioned advantages offer electrospun nanofibers as promising materials to improve wound healing and skin regeneration. The electrospinning method promotes the modulation of the physical and mechanical properties of fibers through tuning the corresponding variables. These superior aspects are the main factors to attend to in designing advanced wound dressings that can mimic the native tissue environment and enhance healing of the wound [3,12,28,50].

### Effect of Electrospinning Parameters on Biological Applications

Electrospun nanofibers may influence and interact with the injured tissue and its biological environment according to their chemical (e.g., composition, degradation), and physical (e.g., diameter, strength, porosity, etc.) characteristics, as well as via additional incorporated bioactive molecules [52]. Hence, the mentioned features of electrospun nanofibers directly affect the efficiency and performance of dressings produced from them. Moreover, the architecture and structure of dressings also significantly affect the wound healing process [5].

There are different parameters and variables that affect the electrospinning procedure and therewith the ultimate characteristics of micro/nanofibers. These factors are generally classified into three categories of inherent solution properties (e.g., polymer concentration, viscosity, solvent system, and conductivity), process conditions (e.g., voltage, distance, flow rate, and nozzle characteristics), and ambient conditions (e.g., temperature, and humidity). All these parameters concur to tune the diameter, morphology, hydrophobicity, thermal and mechanical properties of the electrospun fiber mesh and thereby its possible end-use application as wound dressings and tissue engineering scaffolds [3,33,50]. Hence, proper control of the working parameters and optimization of different electrospinning variables are necessary to obtain structures with the desired physical and biological performance.

**Figure 2:**
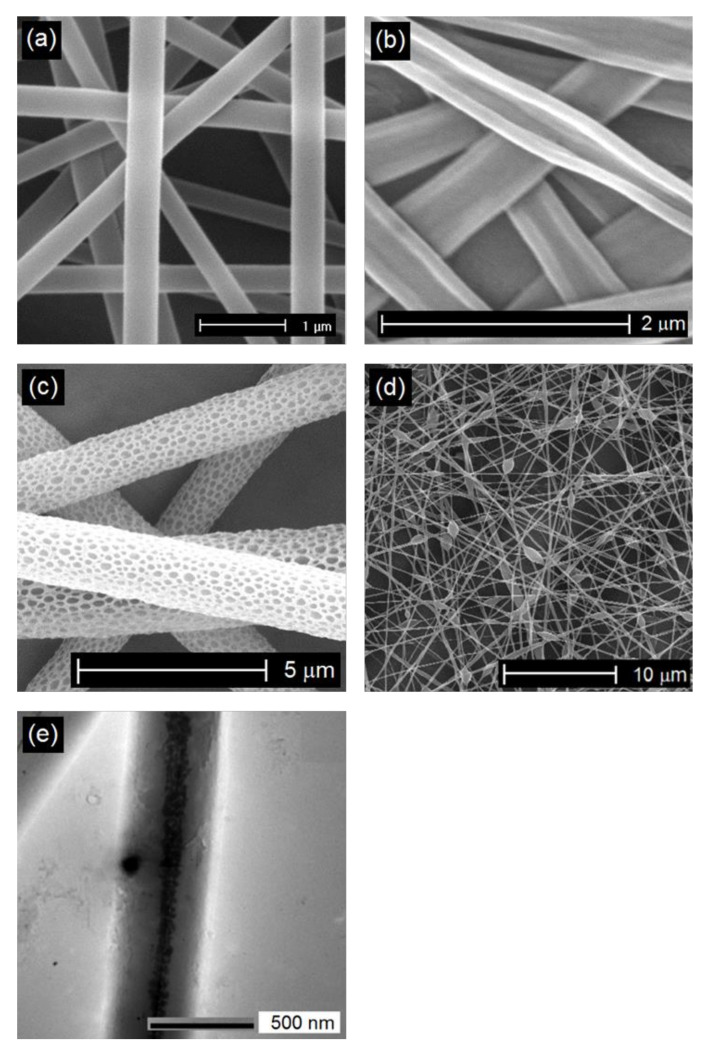
Different morphological structures of fibers can be obtained by altering the electrospinning parameters: (**a**) cylindrical shape with smooth surface (unpublished original picture by the authors), (**b**) flat ribbon like morphology (unpublished original picture by the authors), (**c**) porous (unpublished original picture by the authors), (**d**) bead-on-string morphology (unpublished original picture by the authors), and (**e**) core-shell structure (reproduced from open access article [63] under the terms of the Creative Commons Attribution-Non Commercial License.) All these morphologies are obtained through the electrospinning of polylactide.

**Figure 3:**
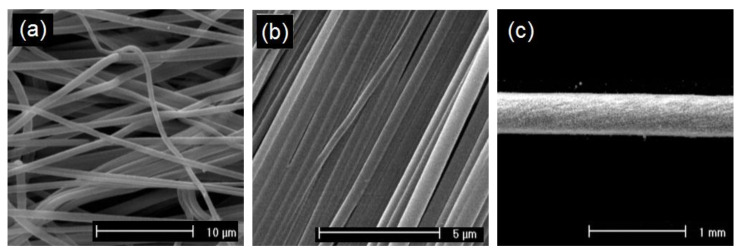
Different arrangements of fiber deposits obtained through electrospinning: (**a**) random, (**b**) oriented, and (**c**) yarn. Unpublished original pictures by the authors. All these structures are obtained through the electrospinning of PLA (original images from the authors).

Different morphological structures (e.g., cylindrical or a ribbon shape, porous, beaded, hollow, and core-shell) (Figure 2) and arrangements (e.g., random, oriented, aligned bundle, yarn) (Figure 3) of fiber deposits can be obtained by altering the electrospinning parameters.

Generally, research attempts are focused on finding the optimized electrospinning conditions to produce uniform structures composed of cylindrical shape fibers with smooth surfaces and without any bead formation (Figure 2a). However, it has been reported that the release of a drug from electrospun fibers with flat ribbon morphology (Figure 2b) might occur through diffusion along the shorter route, due to the high surface area/volume ratio. Compared to flat ribbon shape, cylindrical fibers provided more sustained release behavior [53,54]. Hydrophobicity of fibrous structures, mainly determined by surface topology, is important for their performance in the biological interface, such as drug release, cell adhesion, and proliferation. It has been shown that the flat ribbon-like morphology, restricts the volume of trapped air at the fiber-water interface, and thus affects the surface hydrophobicity [54,55]. Furthermore, when compared to flat ribbon-like shapes, cylindrical morphologies have facilitated cell growth on the membrane surface [54,55].

Since the efficiency of the wound dressings mainly depends on their surface topography, when the fiber morphology changes from a smooth solid structure to a porous one (Figure 1c), several features such as specific surface area, network porosity, and functional versatility may improve [56]. The increased network porosity may provide sufficient space for cell penetration and thus enhance their potential as engineering scaffolds. The porous morphology of the electrospun fibers with small pore size can enhance hemostasis, and effectively protect the wound from bacterial permeation [56]. Those with tiny pores and increased surface to volume ratio can also influence the release behavior of the loaded biomolecules in the fibrous structures [57,58,59]. Although beadless fibers are usually preferred to improve the uniformity of the electrospun nanofibers, bead-on-string nanofibers (Figure 2d), with suitable control of the bead diameter, shape, and surface morphology, have provided efficient encapsulation of biomolecules and controlled release suitable for tissue engineering and wound dressing applications. In vitro release studies have demonstrated that bead- on-string morphology has resulted in a more sustainable release profile with less initial burst release compared to uniform fibers [60,61,62]. Coaxial electrospinning, as an interesting and efficient technology to produce core-shell, has led to several types of researches in the biomedical field (Figure 2e). A common use of this technology is to encapsulate biomolecules in the core part. In this case, the sheath protects the unstable biological agent from an aggressive environment and delivers it in a sustained way by minimizing the burst release. Superior mechanical properties, and the possibility to functionalize the surface without affecting the core material are other advantages [6,59,63,64].

Besides the morphological features, the architecture of the wound dressings and arrangement of the fibers in their structure also greatly affect the adhesion, proliferation, and penetration of the cells, and release behavior of the bioactive molecules from the structure. By applying different collection devices or by manipulating the electric field during the electrospinning process, fiber deposition can be obtained randomly (Figure 3a), up to uniaxially oriented (Figure 3b). Electrospun scaffolds formed from randomly oriented fibers allow adhesion and proliferation of cells in a random way all over the scaffolds. Despite that, on the aligned structures with fibers deposited in a specific direction, cells adhere and grow in the path of the fiber orientation. Aligned fiber deposition can also control the release of entrapped biomolecules by altering the network porosity [3,11,33].

In addition to the abovementioned features, the mechanical properties of the wound dressings and scaffolds are important and can significantly regulate the arrangement of the fibers in the structure. The tensile strength of the dressing should be sufficient to withstand the dressing handling and exchange during the treatment. In biomedical engineering, primary research has focused on the collection of randomly oriented fibrous webs during the electrospinning. Aligned nanofiber bundles preserve more lateral interaction and friction between fibers with highly improved mechanical properties in compression with nanofiber webs. These interactions can be further enhanced by twisting the nanofibers to form yarns (Figure 3c). Aligned fibrous bundles or twisted yarns have developed feasibilities to design a new generation of medical textiles in various wound healing applications, including woven wound dressings, scaffolds for tissue regeneration, and surgical sutures [4,58,65,66].

## 4. Multifunctional Wound Dressings

In recent years, researches have been conducted to develop multifunctional dressings which can supply all the requirements for effective wound healing [67]. The multifunctional composite scaffolds can be produced by blending various natural or synthetic polymers and incorporating drugs, nanoparticles, and bioactive agents through the electrospinning process. More recently, the next generation of multifunctional scaffolds has been fabricated via electrospinning of smart materials [2,5]. Smart materials enable their physicochemical properties to be changed managed by external stimuli such as pH, heat, light, and electric field. These types of materials are interesting for wound healing applications because they have demonstrated multiple advantages compared to ordinary materials [2,11].

### 4.1. Drug Loaded Electrospun Wound Dressings

Multifunctional wound dressings usually need controlled, on-demand release of therapeutic agents, in order to boost restoration within the wound site and prevent undesirable effects such as infection. In this regard, electrospinning offers a great opportunity to prepare drug delivery systems, as it proposes different strategies for incorporating drugs and other biomolecules. Electrospun fibers can deliver agents to the target sites, while reducing the toxic side effects of drugs. Electrospun drug carriers improve the effectuality of drug therapy by controlling the rate, and mechanism of release [58,68,69,70]. Especially, the stimuli-responsive drug delivery systems are promising materials for generating smart wound dressings. These materials, with the ability to tune drug release in response to stimuli of pH [71,72,73,74], temperature [71,75,76], light irradiation [11,75], electric field [77], and oxygen [11,78] hold remarkable promise for wound healing.

**Figure 4:**
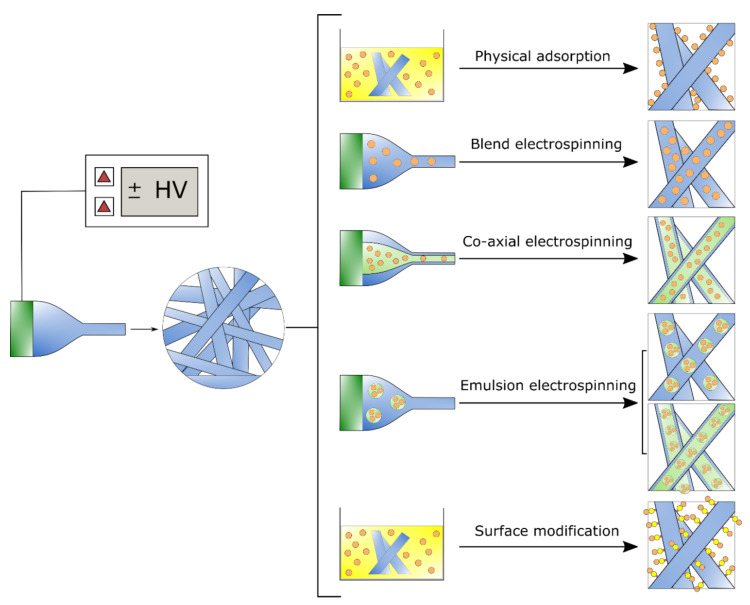
Fabrication techniques of biomolecule-loaded electrospun fibers.

Electrospun fibers demonstrate many advantages for the delivery of biomolecules for healthcare. Owing to the small diameter and large surface area to volume ratio, electrospun fibers grant good drug encapsulation efficiency and result in controllable drug delivery to the on-demand sites [58,68,79]. Furthermore, electrospinning affords various choices by altering the morphology, diameter, porosity, and alignment of fibers by controlling the operational variables and type of materials. Tuning these features, provides modulation of drug release behavior and its kinetics [58,68,80]. For example, it has been reported that the drug-loaded dressings composed of fibers with a smaller diameter and higher surface area showed faster release. Higher porosity of the fibers and structure also causes an increase in the release rate. Fiber alignment in the matrix is another parameter influencing the release behavior through regulating the structure porosity. By electrospinning of different materials with diverse properties, varying polymer blend composition, or altering the percentage of amorphous and crystalline segments in copolymers, degradation properties and release behavior can also be controlled [58,68]. Different strategies, including physical adsorption, blend, or coaxial electrospinning, and surface immobilization have been employed to load the bioactive molecules into the electrospun structures (Figure 4).

Physical adsorption occurs when the electrospun fibers are immersed in a drug-containing solution bath. This strategy is the simplest process to load the bioactive molecules, since the agents inside the bath are inclined to adsorb at the fiber surface. However, its application is limited owing to an uncontrollable release rate [32,43,68,81]. Blend electrospinning is also a simple process to produce drug-loaded fibers by blending the biomolecules with the polymer solution used for electrospinning. Compared to physical adsorption, this method enables the incorporation of the active agents inside the fibers, and thus offers a more sustained drug release profile with an initial burst release of the agents near the surface of the fibers [46,58]. The biomolecules can be completely entrapped in the fibers through the coaxial electrospinning. The release of the agents from core-shell fibers is sustained and burst release is significantly lower than that obtained using blend electrospinning. In fact, by applying this method, the biomolecules loaded in the core layer are well protected by a sheath [63,82,83]. Blend or coaxial emulsion electrospinning is another approach to encapsulate water-soluble agents [9,32,43]. Coaxial and emulsion electrospinning give rise to well controlled release rate and are able to avoid the initial burst release. Surface immobilization (usually, covalent immobilization) enables the attachment of the biomolecules on the surface of the fibers by a chemical bond. Through this method, the surface features of fibrous membranes may be modified [68,84,85].

### 4.2. Electrospun Wound Dressings with Antibacterial Activity

Wound infections are a major global concern and designing antibacterial products for wound healing applications is a prominent field of research [2,63]. In order to prevent deleterious effects caused by infections in the injury area, it is necessary to use a wound dressing capable of both barricading bacterial penetration and microbial colonization into the wound site and supporting skin regeneration [9,50]. The developed electrospun scaffolds with antibacterial activity can prevent wound infection. The antibacterial nanofibers are commonly fabricated by incorporating antibacterial agents during electrospinning. Diverse antimicrobial agents (e.g., antibiotics, metallic nanoparticles, and natural extracts derived products) have been embedded into electrospun nanofibers to improve their antibacterial properties. Metallic nanomaterials such as silver nanoparticles (AgNPs) are known as efficient agents for the treatment of wound infections. Nanoscale particles with high surface to volume ratio, afford a significant improvement in antibacterial activity of electrospun wound dressings [3,63,86,87]. Recent strategies rely on using polymers with intrinsic antibacterial activity, due to physical, chemical or morphological cues which cause an obstacle for bacterial colonization and biofilm formation [88]. Bio-based and biopolymers can offer great opportunities for this purpose.

### 4.3. Electrospun Wound Dressings Loaded with Bioactive Molecules

The incorporation of biological molecules such as growth factors, vitamins, and anti- inflammatory molecules into electrospun fibers is another promising approach to design multifunctional dressings for improving wound healing and skin regeneration [32,89].

Growth factors are biologically active polypeptides that are beneficial to control cell growth, proliferation, and migration during the wound healing process. All the stages of the wound healing process can be regulated using a wide variety of growth factors [90,91]. The role of vitamins in the healing procedure is also considerable. The incorporation of vitamins, particularly vitamins A, C, and, E into electrospun wound dressings aimed at improving healing has been reported in several research works [89,92]. Vitamin A with its proven inflammatory effects can incite angiogenesis and collagenization to enhance wound closure. Vitamin C acts as an antioxidant and accelerates healing through promoting collagen synthesis, acting as an antioxidant, and regulating immune function. Vitamin E relieves free radical detectives in the inflammatory phase of the wound healing process [9,46].

## 5. Application of Bio-Based Electrospun Fibers in Wound Healing

**Figure 5:**
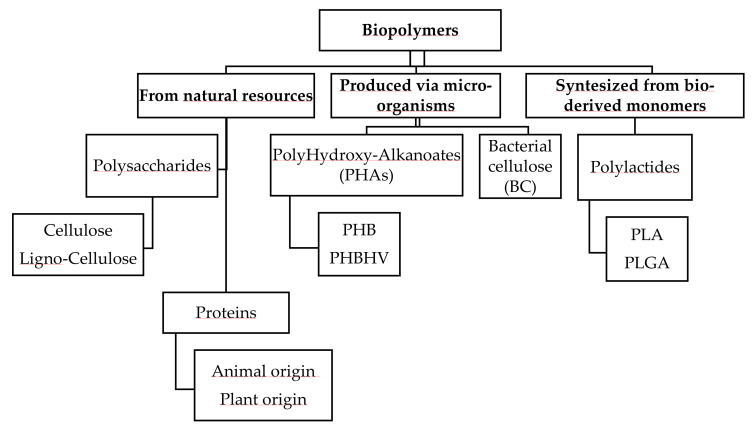
Flow chart showing biopolymers according to diverse origins and types.

Bio-based polymers (also referred to as biopolymers) are organic macromolecules synthesized by living organisms. Biopolymers from different sources such as plant (cellulose, lignin), animal (collagen, chitin, chitosan), micro-organisms (bacterial cellulose, PHA) and biotechnological process (polylactides) (Figure 5), have shown promise in biomedical applications, including drug delivery, tissue engineering and wound healing because of their specific properties. Many of them possess antibacterial, antifungal, antiviral, non/low-immunogenic, renewable, biodegradable, and biocompatible characters [88]. These polymers are expected to soon replace plastic goods in the biomedical sector, not only as proper bioactive devices, but also as sterile packaging and consumables [93,94]. The latter, after inactivation treatments, can indeed provide a better end-of-life option to incineration of biohazard disposables in hospital settings. In addition, wound care products can be developed from biopolymers using different nanotechnology strategies [95]. Biopolymers can be made into fibrous scaffolds in the pure form or blended with other polymers which makes them good candidates for skin substitutes.

### 5.1. Application of Cellulose-Electrospun Nanofibers in Wound Healing (Including Its Composite)

**Figure 6:**
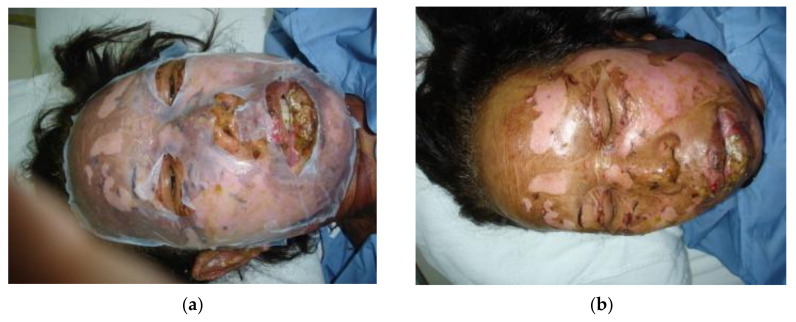
(**a**)The application of a bacterial cellulose-based scaffold as a biological dressing on burned facial skin and (**b**) complete epithelialization obtained after two weeks by using bacterial cellulose (BC) as a temporary skin substitute. Reproduced with permission from [103] (License number: 4875380107950).

Cellulose is a natural, biocompatible, biodegradable, and environmentally friendly, biopolymer which plays an important role in various biomedical applications, including scaffolds in tissue repair and reconstruction, wound dressing, artificial tissue/skin, controlled drug delivery, blood purification as well as cell culture materials [96,97,98]. The moisture-retaining characteristic of cellulose is one of the main reasons for its application in wound care since moist wounds can be treated faster as a result of sufficient growth factors that can be supplied to the healing tissues. The porous structure of cellulose also helps in tissue regeneration via mimicking the skin ECM [99]. Good mechanical properties, high permeability, low toxicity, and adequate conformability are other advantages of cellulose in the application for biological dressings. Using different technologies, such as nanotechnology, biotechnology, three dimensional (3D)- and bio-printing, cellulose properties can be easily tuned to meet the bioengineering demands. For example, different types of nanosized celluloses, especially bacterial cellulose (BC), which is obtained from microorganisms, represent promising functional materials [100,101]. Bacterial cellulose-based scaffolds were investigated in pre- clinical and clinical trials, like wound dressings for skin lesions [102]. To speed up the skin healing of a patient who was suffering with second-degree burns on his face, a bacterial cellulose- based scaffold named Nanocell^®^ was applied and successfully adhered to the wound sites without using other bandages (Figure 6a) [103]. The wounds on the face demonstrated complete re- epithelialization after two weeks (Figure 6b) and during the treatment, no irritation or allergic reaction was observed, indicating the suitability of bacterial cellulose dressings for healing the burned skin. Cellulose nanofibril (CNF) derived from wood has also shown excellent results when used as a biological dressing of skin wounds—it connected well to the wound bed and after skin recovery, separated easily from the surface of the wound itself [100].

**Figure 7:**
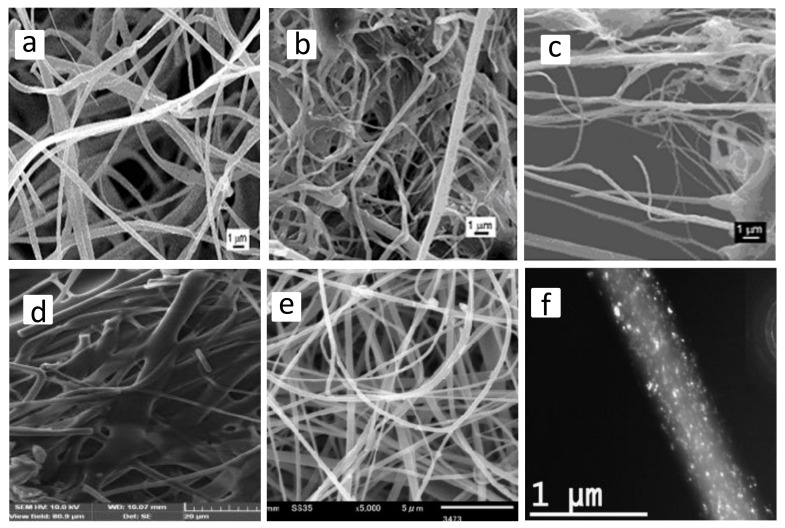
SEM images of (**a**) oxidized cellulose fibers, (**b**) cellulose-chitosan fibers, (**c**) cellulose-poly (methylmethacrylate) (PMMA) reproduced with permission from [38] (License Number: 4873720243859), (**d**) poly (2-hydroxy ethyl methacrylate) (pHEMA)-bamboo cellulose nanocomposite fiber reproduced with permission from [108] (License Number: 4873710894891), (**e**) CA/honey nanofibrous mesh reproduced with permission from [37] (License Number: 4873720705310), (**f**) Transmission electron microscopy (TEM) image of the ZnO/CA composite fiber reproduced with permission from [113] (License Number: 4873720979763).

Electrospinning is an emerging technique, which can also be used to produce cellulose nanofibers or different polymer/cellulose blends or blends of cellulose with nanoparticles with improved functional properties, most importantly, antimicrobial properties in order to avoid wound site infection [104]. High surface areas and extremely interconnected porous structures of nanofibrous nonwovens are naturally appropriate for wound healing application, since they possess a high capacity for exudate absorption and adequate gas exchange [105,106]. Furthermore, cellulose scaffolds are able to carry different bioactive components such as anti-inflammatory and antimicrobial agents [107]. However, only a few studies have focused on electrospinning of cellulose as a control of the solution and electrospinning parameters are required to produce cellulose nanofibers with particular characteristics. The low solubility of cellulose in several solvents, due to strong intramolecular hydrogen bonds is one of the most critical features to be considered [108]. Some solvent systems which can directly dissolve cellulose have low volatility, which is not desirable in the electrospinning procedure [109]. Since cellulose derivatives have better solubility in common electrospinning solvents, about 70% of researches regarding electrospinning of cellulosic materials use cellulose derivatives, such as cellulose acetate (CA) [109]. Liu et al. used blend-electrospinning to produce a series of membranes using polyester urethane (PEU) and CA for wound dressing applications [110]. Hydrophilicity and air permeability of the membrane was improved due to the presence of CA and a humid environment was created, which accelerated wound recovery. A long- term antimicrobial effect was observed for the membranes with controlled diffusion. Electrospun nanofiber composites have also been produced from cellulose blends or derivatives with other biopolymers. Using 1-ethyl-3-methylimidazoliumacetate [EmIm] [Ac] as an ionic solvent, Park et al. [111], developed non-derivatized electrospun chitosan-cellulose composite mesh with satisfactory antibacterial properties, which can be used for treating skin ulcers as a bandage or via incorporation into other absorbents or gauzes. Miao et al. used electrospinning to produce cellulose, cellulose-poly(methylmethacrylate) (PMMA) and cellulose-chitosan fibers (Figure 7a–c) for anti- infective bandage uses [38]. The fibers were functionalized through covalent immobilization of lysostaphin (Lst) and functionalized fibers showed antibacterial activity against S. aureus and low toxicity toward human keratinocytes (HaCaT cells). Electrospun CA/gelatin scaffolds with different CA/gelatin ratio were fabricated by Vatankhah et al. and the best ratios of CA/gelatin for wound care application and for skin regeneration of injured tissues were determined (CA/gelatin l75:25) and (CA/gelatin 25:75) respectively [105]. Roy et al. proved the potential of paclitaxel incorporated poly (2-hydroxy ethyl methacrylate)/bamboo (pHEMA-bamboo) cellulose electrospun fibers as an anticancer structure for coating skin cancers and wound healing (Figure 7d) [112].

Very recently, Ullah et al. successfully fabricated nanofibrous CA meshes (Figure 7e) with different quantities of Manuka honey for potential wound care applications [37]. The high porosity of this nonwoven mesh aided wound breathability, and the presence of honey improved the hydrophilicity of the manufact.

Furthermore, in vitro results have generally shown high efficacy of these fiber meshes in avoiding the growth of bacteria at the wound surface and high cytocompatibility to effectively promote wound healing. Fabrication of cellulose nanofibers containing antimicrobial nanoparticles is an interesting alternative for wound management. Anitha et al. demonstrated that the presence of ZnO nanoparticles inside the CA fibrous membrane improved its antimicrobial properties (Figure 7f)] [113]. ZnO-loaded CA fiber meshes demonstrated antibacterial activity against, Escherichia coli, Citrobacter freundii, and Staphylococcus aureus. Song et al. fabricated cellulose, carboxymethylated cellulose (CMC) and ribbon-shaped CA electrospun fibers, and functionalized their surface with Ag nanoparticles at different pH [114]. Silver nanoparticles covered the surface of fibers according to the following order; CMC > cellulose > CA at the same pH conditions. The presence of nanoparticles finally improved the antibacterial properties and the capacity of CMC fibers for wound healing application.

### 5.2. Application of Chitosan Electrospun Fibers in Wound Healing

**Figure 8:**
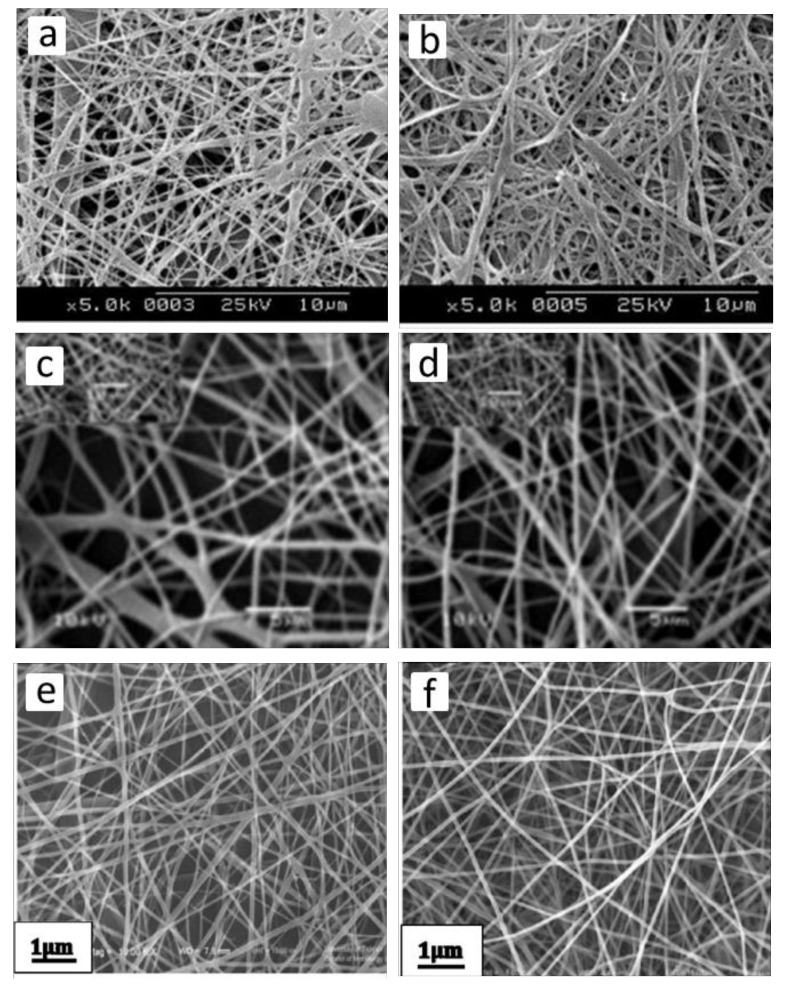
SEM images of (**a**) chitin and (**b**) deacetylated chitin (chitosan) nanofibrous matrix reproduced with permission from [117] (License Number: 4875380936021), (**c**) randomly oriented fibrous mesh of chitosan/polyethylene oxide (PEO) (1:1) (**d**) randomly oriented fibrous mesh of chitosan/PEO/chitin nanocrystals (ChNC) reproduced with permission from [118] (License Number: 4875390863950), (**e**) chitosan/PEO (90/10) nanofibrous matrix, (**f**) chitosan/PEO (90/10) nanofibrous matrix blend with 1 wt% Henna extract reproduced with permission from [119] (License Number: 4875401354853).

Chitin and chitosan; the deacetylated form of chitin are polysaccharides which can be used for wound healing applications because of their antimicrobial, biocompatible, and hemostatic properties [67,115]. Due to the presence of a large number of amino groups on its chain, chitosan behaves as a weak polybase and for this reason exerts antibacterial activity [88]. The pro-inflammatory properties of chitosan have been invoked to play a fundamental role in wound healing procedure. Chitosan is able to accelerate the wound healing process via macrophage activation. Moreover, chitosan is able to develop granulation tissue construction by inducing the migration of polymorphonuclear neutrophils (PMNs) at the start of the wound healing process. Jayakumar et al. demonstrated the potential of chitosan on the regeneration and re-epithelialization of the skin granular layer [116]. Moreover, chitosan proficiently interacts with negatively charged blood cells and with such an efficient adhesion to the wound, it stops the bleeding [88]. Min et al. used electrospinning to fabricate chitin and chitosan nanofibrous matrices (Figure 8a,b) for wound dressings application, using 1,1,1,3,3,3-hexafluoro-2-propanol (HFIP) as a spinning solvent.

However, the applicability of pure electrospun chitosan is limited due to its insufficient mechanical properties [96]. Blend-electrospinning can be used to overcome this limitation [117]. For example, the application of 50 wt% of chitin nanocrystals (ChNC) as a reinforcement in electrospun chitosan fibrous meshes improved tensile strength, modulus, the moisture stability of the as-spun meshes and facilitated water-mediated crosslinking processes. Stable and bead-free random meshes of chitosan/polyethylene oxide (PEO) and chitosan/PEO/ChNC were obtained after spinning for about 30 min (Figure 8c,d). These meshes were cytocompatible toward adipose-derived stem cells after 7 days, and they can be used for wound dressing application [118]. Similarly, electrospun chitosan/sericin [120], and chitosan/silk fibroin [121] composites, showed good antibacterial properties and potential application for wound dressing. In another study, Ardila et al. fabricated electrospun meshes containing chitosan and bacterial nanocellulose using two different approaches including simultaneous spinning of the solutions using two separate syringes and coaxial electrospinning to produce core-shell structures [122]. Coaxial electrospinning led to the formation of nanofibers containing both chitosan and bacterial nanocellulose with a noticeable antimicrobial property which reduced 99.9% of an Escherichia coli population in comparison to the control. As such, it is a promising material for wound healing dressing. Datta et al. developed oleoyl chitosan (OC)/gelatin nanofibrous scaffolds with good mechanical strength, tunable wettability, desirable biocompatibility, and suitable degradation rate for ameliorating full-thickness wound healing in a rat model [123]. Wound contraction and skin tissue regeneration in terms of enhanced collagen deposition and re-epithelialization were significantly improved by the aid of these nanofibers The presence of the long oleoyl tail of OC makes these nanofibers useful for burn wounds, acting as a drug-releasing matrix and at the same time impeding bacterial infection during healing. Different agents including antibiotics, nanoparticles, and/or natural products such as honey and plant extracts have been incorporated within the chitosan electrospun fibers to further improve antibacterial properties, avoid the entry of pathogens into the wound, and kill the invading microorganisms [124]. For example, Yousefi et al., incorporated Henna leaf extract in chitosan nanofibers meshes in order to increase the antibacterial and wound healing efficacy (Figure 8e,f) [119]. Continuous and bead-free nanofibers with a diameter of 64–87 nm were prepared with notable antibacterial activity, and can be considered as biodegradable, biobased, and antibacterial wound healing dressings.

### 5.3. Application of PHA Electrospun Fibers in Wound Healing

PHAs are a family of linear thermoplastic bio-polyesters. They are synthesized by many microorganisms under unbalanced growth conditions, as an alternative nutrient (carbon) reserve [125,126]. Over the last three decades, many studies have investigated the physicochemical properties of this new class of biopolymers, revealing innumerable advantages in using PHAs as a biomaterial in medical applications thanks to their biocompatibility, mechanical stability, strength, and biodegradability under physiological conditions with non-toxic degradation products [127,128,129]. Studies also explored pharmaceutical applications of some degradation products of PHA, finding that they can evoke an inhibitory effect on microbial growth [128,129,130,131]. To date over 100 units of PHA monomers have been identified and no studies have reported carcinogenesis induced by any PHA or their biodegradation products [125,128,129]. The properties of PHAs vary considerably depending on their monomer content and hence can be tailored by controlling their composition [126,132]. Furthermore, PHAs can be surface modified, blended with other polymers, and composite with inorganic materials such as nanoparticles (NPs), nanocrystals (NCs), drugs, and biomolecules to enhance their biocompatibility, antimicrobial activity, mechanical and thermal properties, and degradation rates, depending on the required application [125,128,132].

Electrospinning of PHAs has also been extensively investigated [133,134,135]. This technique enabled a simple, scalable production of ultrafine fibrous meshes, eventually incorporated, and/or blended, by direct mixing in the electrospinning solution with different organic and inorganic materials, to reach the targeted properties [133,136].

There are many advantages in using electrospun nonwoven membranes in wound healing applications, mainly associated with their structural porosity, wettability, and similarity to the natural ECM, which has shown to effectively promote cellular migration, attachment, and proliferation [125,137,138].

Towards the last decade, electrospun PHA meshes started gaining high attention for potential applications as a wound dressing in skin regeneration. A wide range of PHAs have been used to produce electrospun fiber meshes with different morphology and alignment for wound healing application [125,137,138,139,140], but in recent years, most studies have focused on the application of PHA- based blends and composites, or functionalized electrospun fiber meshes with improved physicochemical and bioactive properties [137,139,141,142,143]. For example, Shishatskaya et al. used poly(3-hydroxybutyrate-co-4-hydroxybutyrate) [P(3HB-co-4HB)], considered one of the best choices among the PHAs to produce electrospun fibers for wound healing applications, due to its low degree of crystallinity and high elasticity [137]. They demonstrated that the presence of fibroblasts inside the fibers had a significant effect on the amount of hyperemia and purulent exudate, and considered use of composite fibers a better candidate for wound healing application. The wounds under the cell- loaded P(3HB-co-4HB) membrane showed 1.4 times faster healing with respect to the wounds under the cell-free membrane and a 3.5 times faster healing than the wound healing under the eschar (control). Complete healing was achieved after 14 days in the cell-loaded membrane group while, approximately 90% and 70% area reduction were observed in the pure P(3HB-co-4HB) meshes and control groups, respectively. The use of keratin-loaded-poly (3-hydroxybutyrate-co-3-hydroxyvalerate) (PHBV) electrospun fibrous meshes for wound dressing applications has also been investigated [142]. These fiber meshes were tested for cell viability on NIH 3T3 cells and on a wound closure follow-up, conducted on athymic nude mice, consisting of five-time points (0, 2, 4, 7, and 9 days), using gauze as negative control and pure PHBV ultrafine electrospun mats for comparison. On day-3 and day-5, cell viability on the keratin-loaded PHBV mesh was significantly higher than that of the PHBV control. The dimensions of the wound and histology of the healing tissue were observed, and the results showed that on day-9, only the wound under the keratin-loaded PHBV mesh was almost closed (i.e., 94% wound size reduction) and, in the newly formed skin tissue, complete and uniform re-epithelization of the epidermis was formed. At the same time, only a small epidermal layer was observed in the PHBV mesh and the gauze controls. Some studies explored the influence of blending poly (3-hydroxybutyrate) (P3HB) or PHBV with collagen and/or gelatin on the scaffold properties, cell viability, and/or wound closure tests [139,143,144,145]. In fact, adding a hydrophilic natural protein (e.g., collagen) to pure PHBV (or to PHAs in general) highly increases the scaffold wound exudate absorption capacity and cell-scaffold interactions. In 2007, two different groups demonstrated that the presence of collagen in PHBV electrospun nonwoven mesh significantly improved cell adhesion and proliferation of NIH3T3 and dermal sheath (DS) cells/epithelial outer root sheath (ORS) cells [139,144]. Han et al. further evaluated the effectiveness of these scaffolds in vivo using Athymic nude mice for an open wound-healing model and, interestingly they pointed out a faster wound closure and better healing for pure PHBV mesh than for the blended one. They assessed the result by checking that filaggrin (protein that binds to keratin fibers in epithelial cells) was strongly expressed on the upper epidermal layer of PHBV-grafted skin, while only a little filaggrin was observed on the PHBV/collagen-grafted tissue [139]. According to the better cell viability of PHBV/collagen meshes and better wound closing and healing of pure PHBV meshes, they concluded that the physical property and mechanical stability of the matrices, given by the PHBV component, seemed to be a more important factor for early-stage wound dressings than their cell culture activity.

**Figure 9:**
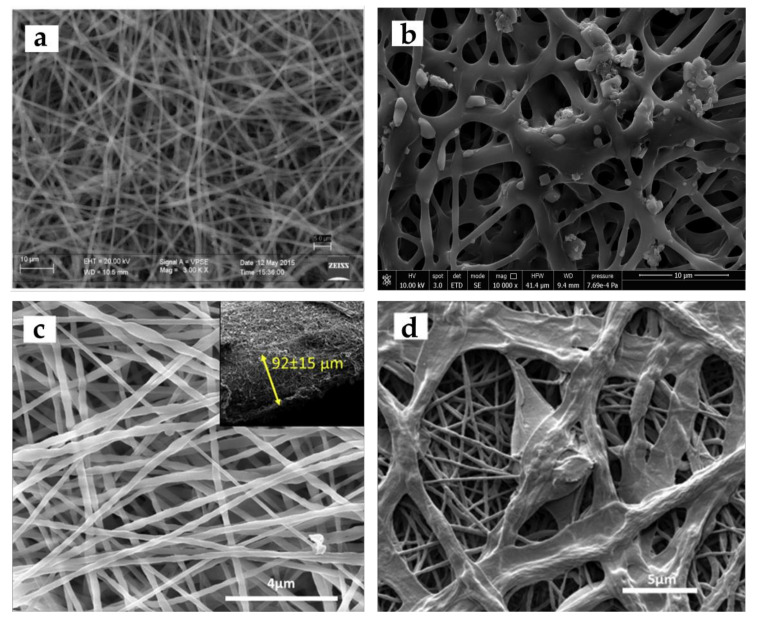
SEM images of (**a**) poly(hydroxybutyrate) (PHB)/Collagen (50/50 wt%) electrospun fiber meshes reproduced from open access article [143] distributed under the Creative Commons Attribution License, (**b**) poly(3-hydroxybutyrate-co-4-hydroxybutyrate) P(3HB)/P(3HO-co-3HD) electrospun fiber meshes functionalized with electrosprayed chitin-lignin/glycyrrhizin acid (CLA) (**c**) poly (3-hydroxybutyrate-co-3-hydroxyvalerate) (PHBV)/Curcumin (0.5) electrospun fiber meshes and cross-sections of drug-loaded nanofibers and (**d**) SEM images of L929 fibroblast cells cultured on PHBV/Curcumin (0.5 w/v%) electrospun fiber meshes after incubation for 14 days reproduced from [39] (License Number: 4876940110497).

Salvatore et al. successfully produced and investigated the potential of electrospun PHB/collagen type I meshes for tissue engineering applications at PHB/collagen weight ratios of 100/0, 70/30, and 50/50 *w/w* (Figure 9a) [143]. The meshes were demonstrated to be effective substrates for viability and proliferation of murine fibroblasts for up to six days of culture at all the three different weight ratio compositions. The introduction of collagen improved the wettability and thermal stability of the scaffold and decreased the crystallinity degree, also enhancing the sensitivity to hydrolytic degradation. The morphological, mechanical, and degradation properties of the obtained meshes were suitable for wound dressing applications and all tunable to some extent by adjusting the PHB/collagen weight ratios. Interestingly, the 50/50 *w/w* sample displayed the highest wettability and degradation rate, while maintaining an elastic modulus comparable to that of pure PHB samples. Overall, although preliminary, the study revealed the possibility of tuning different physicochemical properties of electrospun PHB meshes by blending them with a specific amount of collagen directly in the electrospinning solution, keeping good cell viability and proliferation at all the different ratios.

Azimi et al. used electrospinning in order to produce blended poly (3-hydroxybutyrate)/poly(3-hydroxyoctanoate-co-3-hydroxydecanoate) [P(3HB)/P(3HO-co-3HD)] fiber meshes surface-decorated via electrospray of chitin-lignin/glycyrrhizin acid (CLA) complexes (Figure 9b) [146]. CLA complexes are bio-based micro-compounds that can be considered useful bioactive agents for functionalizing skin contact substrates, as they showed a proficient interaction in an in vitro skin model [147]. These bio-based and biodegradable functional nonwovens showed strong anti-inflammatory activity which is promising in wound healing applications. Kandhasamy et al. developed a composite scaffold for effective wound healing treatment based on a PHB/gelatin/ostholamide (OSA) electrospun blend coated with collagen. The obtained scaffold showed great mechanical stability, stable enzymatic degradation, and efficient antimicrobial activity against Pseudomonas aeruginosa and Staphylococcus aureus due to the sustained release of OSA [145]. In vitro and in vivo analysis displayed excellent cytocompatibility, determined by NIH 3T3 fibroblast proliferation studies, and good wound healing efficacy confirmed by using an open wound model in Wistar rats. In fact, after 15 days, complete wound closure was achieved using the PHB/gelatin/OSA/collagen scaffold, meanwhile wound size reductions of approximately 75%, 65%, and 45% were respectively observed for PHB/gelatin/OSA scaffold, pure collagen scaffold, and the cotton gauze positive control.

Recent studies have shown that many additives (e.g., drugs, NPs, NCs, essential oils, biomolecules) can be incorporated into nanofibers to enhance their mechanical and antimicrobial properties [148,149], and the nanofiber structure enables tunable sustained drug release, which in turn can promote/regulate the wound healing process. The increasing outcome of antibiotic resistance during the last years has also brought a big interest in developing new antimicrobial agents. The antimicrobial activity of some NCs and metal/metal oxides NPs, together with the capability of dispersing them in electrospun matrices, have been extensively studied and reviewed [148,150,151,152,153]. In this regard, recent studies focused on the wound dressing application of these nanocomposites. For example, Abdalkarim et al. reported the potential of cellulose nanocrystal-ZnO nanohybrids (CNC-ZnO) incorporated in PHBV electrospun fibers at different concentrations (3–15 wt%) for antibacterial wound dressings [154]. Results showed that the presence of CNCs-ZnO inside the fibers led to a reduction in fiber diameter and crystallinity and increased the porosity, thermal stability, and mechanical stiffness of the fibrous meshes, especially for 5 wt% CNC-ZnO, with respect to the pure ones reporting an increase of 150% in tensile strength and 112.5% in Young’s modulus. Furthermore, enhanced absorbency capacity of the fresh blood model (4 g/g for PHBV meshes and 8.4 g/g for the membrane with 5 wt% CNC-ZnO), better barrier properties, and excellent antimicrobial activity were observed in CNC-ZnO/loaded PHBV fibers [154,155]. The inclusion of natural bioactive substances into PHA nano/micro-fibers have also been explored. For example, Mutlu et al. incorporated curcumin as an antioxidant, anti-inflammatory, and antitumor agent-into the PHBV nanofibers (Figure 9c) [39]. These matrices showed potential for wound dressing application since they were not toxic towards the L929 mouse fibroblast cell line (Figure 9d). The presence of curcumin particles inside the fibers increased swelling capacity, cell attachment, and proliferation while due to its plasticizer effect, decreased the mechanical properties of the matrix but still provided enough mechanical strength during the wound healing process. Diabetic wounds can often become chronic, with nonhealing mainly associated with insufficient cell proliferation and angiogenesis. Furthermore, reactive oxygen species (ROS) have been shown to delay the healing process, exacerbating the chronicity status; therefore, they cannot be used as antimicrobial agents. On the other hand, antioxidant therapies have been shown to improve the healing of chronic diabetic wounds by inhibiting ROS generation [156,157]. Cerium oxide nanoparticles (nCeO_2_) show effective antioxidant activity, so their application in diabetic wound dressings can be a promising approach to promote angiogenesis and healing. Augustine et al. developed for the first time a novel electrospun PHBV membrane containing nCeO_2_ for diabetic wound healing [158]. They reported that an optimum load of 1% w/w nCeO_2_ can improve the tensile strength of the neat PHBV membrane without a significant loss of elongation at break. They assessed excellent in vitro cytocompatibility and cell adhesion properties, especially for the 1% w/w nCeO_2_ load, and enhanced cell migration and angiogenesis were observed for all the nanocomposites in comparison to pure PHBV meshes. Finally, they demonstrated the efficiency of nCeO_2_ containing membranes in enhancing the diabetic wound healing process on full-thickness excision wound model performed on Male Sprague–Dawley rats [158].

Electrospun PHBV fibers have shown the possibility of being incorporated in hydrogels, thus generating a fiber-based composite material apt for controlled release of drugs [159]. Cristallini et al. proposed this method for generating a surface coating able to release dexamethasone, thus modulating the fibrotic encapsulation of implanted devices.

Finally, we would like to report (even if not fully biobased) a smart exploitation of the hydrophobic nature and the specific biotic enzymatic degradation mechanism of some PHAs. Specifically, Liu S. et al. used a blend composed of PHBV/polyethylene succinate (PES)/poly(3-hydroxyoctanoate-co-3-hydroxyhexanoate) (PHOHHx), in a ratio of 3:2:1 (*w/w*) to produce core-shell fibers which can effectively bring on a burst release of antibiotic triggered by bacterial presence. The use of broad-spectrum potent biocides can play an active role in the wound healing process by preventing or treating infections but, on the other hand, its use can develop bacterial resistance against antibiotics and attack pathogens and host cells non-selectively, further delaying the wound healing process. For these reasons, core-shell ultrafine fibers were developed using coaxial electrospinning, applying the mentioned blend as shell, and a core consisting of polyvinylpyrrolidone (PVP) as a polymer carrier and dodecyl trimethyl ammonium chloride (DTAC) as a model biocide. In vitro results showed that this composite structure effectively prevented degradation of the PHAs/PES based shell in abiotic solutions, with slow releasing rates of the biocide. An opposite trend was observed in the presence of the model bacteria Pseudomonas aeruginosa. The in vitro release data demonstrated the bacteria-triggered drug delivery property of PHAs/PES based core-shell nanofibrous membranes [160].

### 5.4. Application of PLA-Based Electrospun Fibers in Wound Healing

PLA is a synthetic biopolymer made from renewable resources. PLA can be derived from natural raw materials such as corn starch, rice, and sugar cane through ring-opening polymerization or condensation polymerization of the lactic acid. It has been approved in diverse applications by the Food and Drug Administration (FDA). Lactic acid (2-hydroxypropionic acid, CH3–CHOHCOOH) is the constituent monomer of PLA. Poly (l-lactide) (PLLA), Poly (d-lactide) (PDLA), racemic poly(dl-lactide) (PDLLA) are three isomeric forms of PLA [51,69,161].

PLA is a biocompatible, biodegradable, and bioabsorbable aliphatic thermoplastic polyester with valuable thermo-mechanical properties. These features, in addition to its non-toxicity to the human body, make it a suitable candidate for use in bioengineering [6,26,31,162]. In some applications, such as medical implants, PLA stereocomplex has attracted growing attention. PLA stereocomplex can be obtained by the strong interaction formed by the side-by-side arrangement of the molecular chains of enantiomeric PLA polymers such as PLLA and PDLA. The formation of stereocomplex also endows PLA-based materials with enhanced mechanical function, thermal stability, and hydrolysis resistance [163,164].

Electrospun nanofibers based on PLA have been widely considered for biomedical applications as tissue engineering scaffolds, wound dressings, and drug carriers [7,165]. The related researches are briefly reviewed in this section, with a focus on different aspects of the electrospinning process of PLA, biomolecule-loaded PLA-based nanofibers, and clinical applications of the electrospun PLA- based structures as wound healing materials.

The electrospinning process of PLA has broadly been discussed; a lot of research efforts have been focused on the systematic investigation and optimizing the parameters that influence the ultimate properties of electrospun PLA fibers and thus their possible end-use. Depending on the characteristics of the PLA solutions, the process parameters and ambient conditions, the diameter and morphology of the PLA fibers produced by electrospinning will differ. As an example, in research works performed by Maleki et al. the influence of the solvent system on the electrospinning of PLLA fibers is investigated [57,66]. The results demonstrated that the dielectric constant and vapor pressure of the solvent are important parameters, which significantly affect the fiber morphology, diameter, and crystallinity and hence influence the mechanical properties of the fibrous structures. Electrospinning of PLLA solutions using solvents with high vapor pressure and lower boiling points, like chloroform and dichloromethane, formed fibers with porous surfaces. Whereas the electrospinning of PLLA solution based on solvents such as hexafluoro isopropanol (HFIP) and 2,2,2- trifluoroethanol (TFE) let to formation of uniform fibers with a smooth surface (Figure 2a) [51,57,66]. Solvents with a low vapor pressure like 2,2,2-trifluoroethanol (TFE) provided enough time for crystals to grow during fiber formation resulting in a high crystallinity. Solutions obtained from chloroform with lower conductivity caused less stretching of the charged jet in the electrostatic field and resulted in larger diameters of fibers [57,66]. In another study by Maleki et al., in order to investigate the effect of solvent type on the mechanical properties of electrospun PLLA structures, a double nozzle electrospinning set-up was used to fabricate continuous twisted yarns from PLLA fibers. The tensile strength and modulus of electrospun yarns produced from a solvent with low vapor pressure like TFE showed higher tensile strength and modulus than yarns produced from fibers electrospun from PLLA-dichloromethane or chloroform solutions [66]. The PLA-based solution properties like concentration and viscosity are the other factors that affect the ultimate characteristics of the electrospun PLA fibers and have been considered by several researchers. It was shown that, by increasing the solution concentration, uniform fibers were produced with growing average diameters [57,65].

The applications of electrospun PLA-based structures for wound healing and skin regeneration have also been investigated in several recently published research works. Most of these efforts were made towards the development of bioactive PLA-based electrospun membranes for the designing of wound healing products with multifunctional properties. In this context, electrospun PLA fibers were employed as drug delivery carriers.

Alves et al. recently confirmed that PLA electrospun membranes are promising drug delivery systems for sustained release for developing wound dressings [69]. They employed both techniques of physical adsorption and blend electrospinning to load anti-inflammatory agents, dexamethasone acetate (DEX), and betamethasone to PLA electrospun fibers and compare their release efficiency. The influence of both drugs on the features of the electrospun PLA fibers, such as morphological and mechanical properties, was studied. The drug-loaded fibers produced from the blend electrospinning of the PLA solution containing drugs presented a better sustained release profile after a burst release during the first five hours. Samples prepared by physical adsorption of the drug on the PLA electrospun membranes showed a significant burst release.

Pankongadisak et al., developed electrospun curcumin incorporated PLLA fibers as carriers to control curcumin release with anti-inflammatory, and antioxidant properties for wound dressing materials. The fabricated membrane through blend electrospinning was non-toxic to human adult dermal fibroblast (HDF) cells and protected cell attachment and proliferation [166].

In a research attempt performed by Moradkhannejhad et al., the blending of PLA and poly(ethylene glycol) (PEG) aiming to increase the hydrophilicity of PLA for wound dressing applications is reported. In their study, the hydrophobicity of curcumin (CUR) loaded PLA electrospun nanofibers was modified through the addition of PEG with different contents and molecular weights [31]. The incubation of PLA/CUR/PEG nanofibers in PBS indicated that the increase in concentration and a decrease in the molecular weight of PEG caused an increase in the weight loss values. The prepared electrospun CUR-loaded PLA/PEG membranes provided desirable conditions for cell growth and to accelerate the drug release through balancing the hydrophilicity– hydrophobicity of the medium.

In order to improve the elasticity and hydrophilicity of the PLA nanofibrous membranes as wound dressings, in a study by Zou et al., PLA/Poly (1, 8-octanediol-co-citric acid) (POC) nanofibers were fabricated using the blend electrospinning method. Aspirin loading and its release behavior were also investigated. In these authors’ view, the developed elastic, hydrophilic, and biodegradable PLA-based nanofibrous membrane can modify several disadvantages of PLA dressings, namely, limited tensile deformation, uncontrollable degradation rate, and low hydrophilicity, which hinder its application in wound dressings [167].

Owing to its biological features, PLA is often used as the main component of electrospun composites for biomedical applications. In a research study, by Bi et al., PLA and PLA/PVA/SA (sodium alginate) membranes were produced via electrospinning for wound healing applications. The obtained results suggested these membranes could be used as novel wound dressings. In vitro experiments indicated that PLA and PLA/PVA/SA electrospun membranes were able to provide good support for the growth of mouse fibroblasts (L929 cell line). The fibroblasts displayed relatively better adhesion and proliferation on PLA/PVA/SA than on the PLA r membranes. The in vivo assay also confirmed that the PLA and PLA/PVA/SA electrospun membranes significantly enhanced wound healing compared to commercially available gauzes [26]. Ilomuanya et al., designed PLA and collagen-PLA electrospun fibrous scaffolds with antibacterial activity for wound healing applications [12]. They used silver sulphadiazine (Ag+S) and Aspalathus linearis (AL) fermented extract to improve antibacterial properties and cellular biocompatibility. They were nontoxic to the cells and provided favorable substrates for cell attachment and proliferation. In another study by Cui et al., doxycycline (DCH), a broad-spectrum antibiotic, selected as a model drug, and DCH-loaded PLA nanofibers were produced by the blend electrospinning process [42]. Results of cytotoxicity and antibacterial tests revealed that DCH-loaded PLA nanofibers showed favorable cytocompatibility to L929 mouse fibroblasts and exhibited good antibacterial activity, suggesting its potential as wound dressings for chronic wound healing. With the purpose of avoiding disadvantages of conventional antibiotic-loaded wound dressings, Zhang et al. in their research work employed the electrospinning process to develop fibrous composite based on silver (I) metal-organic frameworks-PLA antibiotic- free wound dressing with the efficient antibacterial capability to promote tissue regeneration, simultaneously. The in vivo experiments indicated that the electrospun fibrous composite could significantly accelerate the healing rate of infected wounds in rats [10]. Fang et al. employed the coaxial electrospinning method to develop core-shell nanofibers based on PLA and γ- PGA for wound healing as tissue engineering scaffolds. The in vitro cell culture study and in vivo animal experiment on PLA/γ-PGA core-shell nanofiber membrane demonstrated favorable biocompatibility with good ability to wound repair [6]. A study by Yang et al. used poly (glycerol sebacate) (PGS)/PLLA fibrous scaffold with a PGS core and a PLLA shell via coaxial electrospinning. The fabricated fibers with porous morphology of the shell surface showed excellent ability to repair tissues of the skin wound. In comparison to pure PLLA scaffold, the core-shell structure exhibited superior cell proliferation, with a lower inflammatory response [51]. In another work performed by Yang et al., the coaxial electrospinning technique was applied to fabricate the core-shell structured PLLA/chitosan nanofibrous scaffolds for wound dressing applications. In this study, graphite oxide (GO) nanosheets were coated on core PLLA-shell chitosan nanofibers to provide a synergistic microenvironment for wound healing. The coating with GO nanosheets significantly enhanced the hydrophilicity of the electrospun membrane. GO coated chitosan/PLLA nanofibrous scaffolds indicated desirable antibacterial activity. In addition, they promoted the growth of pig iliac endothelial cells (PIECs). GO-coated chitosan/PLLA nanofibrous scaffolds possessed favorable wound healing in rats [168]. In a research work performed by Augustine et al., the wound healing membranes composed of core-shell fibers were produced by coaxial electrospinning. Connective tissue growth factor (CTGF) was encapsulated within the PVA core which was covered by a thin layer of PLA. The developed CTGF loaded core (PVA)-shell (PLA) membranes boosted its sustained release for diabetic wound healing applications [165].

The effect of scaffold architecture on healing efficiency was lately investigated by Majchrowicz et al. [169]. In this work, a nanofibrous composite was made of PLA matrix and calcium phosphate organically modified glass (CaP ormoglass) nanoparticles fabricated by blend electrospinning having two arrangement of the aligned and random orientation of fiber deposits. The addition of CaP ormoglass nanoparticles in electrospun composites promoted bone regeneration by improving the degradation process. Random fiber orientation seems to be superior for CaP compounds released during in vitro degradation. Differently, for their aligned architecture only small amounts of CaP deposits were observed after 21 days of immersion in PBS. Recently, a wet electrospinning technique with a liquid coagulation bath collector was applied in order to produce 3D porous PLA nanofibrous scaffolds to seed rat bone-marrow stem cells (BMSCs). Wet electrospun nanofibers have distinguished advantages such as high porosity and surface area compared with the dry electrospinning technique. The results of in vitro and in vivo assay proved that the 3D electrospun fibrous PLA can be a suitable dressing for wound repair [40].

## 6. Translational Approaches

### 6.1. Drug Delivery Electrospun Fibers

Controlled drug delivery systems (DDSs) display one of the advancing areas of biomedical and pharmaceutical sciences [170]. In biodegradable polymers, the drug release system encompasses a faster drug desorption from the surface and a slower drug diffusion from the bulk, which is accelerated during polymer degradation. The velocity of these phenomena also depends on the surface to volume ratio and chemistry of drug versus polymer. Some unique advantages of electrospun fibers including impressive drug loading and encapsulation, easy modulation of the release rate, and its simple processability aid DDSs to achieve controlled drug release. Electrospun nanofibers can be used for transdermal drug delivery systems (TDDS), in which target agents and sensitive drugs, which cannot be transferred orally are delivered through the skin into the body. Indeed, different herbal pharmaceutical compounds (e.g., aloesin, curcumin, thymol, etc.) can be incorporated in electrospun nanofibers in order to expand TDDS and/or active wound dressings. The release mechanism understanding is essential for developing the DDS systems. Mainly release mechanisms are diffusion (through water-filled pores of the polymer bulk), degradation/erosion, and osmotic pumping.

Single-nozzle electrospinning produces fiber meshes which usually act as degradation-based DDSs since the drugs are dispersed/dissolved within the dissolvable/degradable polymeric matrix in which dissolution or degradation of the polymeric matrix leads to drug release. Degradation occurs in the bulk since the average molecular weight of polymer decreases while erosion occurs at the surface when the polymer undergoes a decrease in total mass [171]. On the other hand, a reservoir DDSs can usually be produced by coaxial electrospinning in which the drug molecules are encapsulated in the inner solution core, surrounded with the outer shell barrier, and their release profiles are usually governed by diffusion through the shell membrane which helps to delay fast drug diffusion. The shell thickness and core diameter play an important role in the extent of the drug release delay [172].

While prior approaches are based on the encapsulation of drug molecules within the nanofiber bulk phase, surface-modified electrospun fibers have also opened up a new possibility of constructing more complicated drug delivery platforms. Indeed, premier adhesiveness toward biological surfaces lead nanofibers to be a suitable nominate for topical drug delivery devices [173]. Physical immobilization of drug molecules on the surface of nanofibrous meshes with high surface area to volume ratio leads to higher drug loading amount per unit mass in comparison to any other device. For some particular applications in which prevention of bacterial infection is required, the instant release of drugs from the surface of the nanofiber is advantageous since it can provide facile dosage control of some specific pharmaceutical agents [174]. Furthermore, some specific structures such as drug-loaded nanoparticles located on the surface of nanofibers in addition to the high drug loading capacity are able to provide unique drug-releasing profiles, which the nanofiber itself cannot obtain [175].

Table 1 summarized the use of different biopolymers, incorporating or not active ingredients, to have a specific function and target specific wound types, which include the types chronic/acute, localized/extended, infected/inflamed, due to burning, diabetes, ulcers and trauma. It can be observed that the most searched features in wound dressing function rely on antimicrobial and anti- inflammatory properties to allow efficient wound healing in complicated wound types. Using conventional wound dressings, the most frequent side effect reported is dermatitis, especially in patients with leg ulcers [176]. Allergic contact dermatitis is an individual host response to a material, hardly predictable. However, a careful choice of biomaterials, in vitro and preclinical investigation on immune response may help in developing better biocompatible wound dressings.

### 6.2. Clinical Trials of Electrospun Wound Dressings

Despite the great number of produced and ongoing researches highlighting the potential of electrospun meshes as wound dressings and, more in general, for pharmaceutical applications, an actual impact in the clinics with any therapeutic product for use in humans has not yet been achieved [177]. Among the many proposed products, a few have reached an actual preclinical level [178] and very few an in-human clinical trial [177,179,180]. Of three of these products, regarding wound dressing applications, namely SurgiCLOT^®^ [181], Pathon [182,183], and TPP-fibers (Tecophilic™) [184], to our knowledge, only SurgiCLOT^®^ is currently available on the market. Table 2 reports the in-human tested and commercially available products related to the electrospinning technology for wound dressing. In this table, we also reported SpinCare^TM^, which is a portable device for electrospin directly onto the wound. It has also to be noted that actually, only SurgiCLOT^®^ made from dextran, a polysaccharide, is purely bio-based. Dextran is the collective name given to a large class of α1→6- linked glucose polymers. It is synthesized from sucrose by certain lactic acid bacteria, such as *Leuconostoc mesenteroides*, *Lactobacillus* spp., and *Streptococcus mutans*. Hydrogels of this polysaccharide are particularly suitable as scaffolds for soft tissue engineering applications because dextran is resistant to both protein adsorption and cell adhesion. Incidentally, this biopolymer is used in the clinics for its antithrombotic (antiplatelet), properties. Polyurethanes are a class of synthetic polymers. However, one of the special features of polyurethanes is that their monomers can be derived from natural sources. The principal natural source of polyols are vegetable oils, like castor oil, olive oil, and canola oil. Thus, it may be expected that green polyurethanes will enter into biomedical products in the future. The biodegradation of polyurethanes in the physiological environment is complex, but involves three main mechanisms: hydrolytic, enzymatic, and oxidative. The degradation products depend strongly on the chemistry of the polymer but also on the degradation mechanism.

Mulholland, E.J., in his recent work presented great perspectives in using electrospun meshes for scar treatment, highlighting the clinical absence of any effective electrospun anti-scarring device [179]. He also reported that there are currently five clinical trials exploring the use of electrospun nanofibers scaffolds, but no current recruiting trials [192].

The problems that have slowed down the translation of clinical electrospun nanofiber products into the market have been associated with two main different aspects: the first is related to the manufacturing and technical part. In fact, although highly scalable, the industrial throughput is (still) not competitive and there is a need for highly skilled workers to produce and develop new products [193]. In biomedical applications, due to the reduced area of the devices, this problem seems more easily solvable. The second aspect is the difficulty to achieve the legal standards posed by the regulatory bodies for these novel technologies which exploit state-of-the-art research, such as the sterilization part and the application of devices containing living cells and/or bioactive molecules [177,179,194].

## 7. Future Perspective

Wounds have been affecting people of all genders, race, and age throughout the history of humankind. The integration of textile technology and medical science presents a new field known as “medical textile” which is a substantial and growing section of the textile industry. The noticeable advancement of biomaterials science, especially in the field of bio-nanotechnology, will generate a great number of opportunities for medical textiles in the coming decade. Wound dressings are an important component of the “on the patient” medical textiles [195], as effective wound dressings can convert painful days to comfortable days for patients. Since the nineties, several biomaterials and technologies have been developed for producing wound dressings. As such, the numerous research efforts in this field performed by research institutes and industries around the world, are expected to have a considerable effect on the quality and expectation of life.

The market for bioactive wound care products is growing fast, as various biomaterials, superabsorbent and multifunctional dressings are continuously being proposed instead of conventional gauzes. Multiple and specific functionalities, such as antimicrobial, hemostatic, bio- responsive and biomimetic properties are required for future wound care products to provide a suitable micro-environment. Unveiling chemical, physical, and mechanical properties of existing biopolymers and even designing new ones, would push forward the availability of the desired efficacy in wound dressings. To this purpose, the recent advancements in regenerative medicine, nanotechnology, and bioengineering technologies, allow the development of specific key structures for wound healing applications. Electrospinning and bioprinting enable the development of 3D biopolymer-based scaffolds and 3D artificial skin mimicking with the desired pore size and tensile strength. In particular, electrospinning enables the production of multifunctional nanofibrous materials, which not only provide functionalities such as physical protection and optimal moisture environment for the wound, but also permit a sustained release of the therapeutic agents to provide the requirements of the healing procedure [196]. These functional materials should be non-toxic and provide comfort for the patient through gas permeability, and easy removal. More advanced needs, such as microbial control can accelerate the wound healing process. A suitably designed dressing should provide appropriate warmth in cold weather and coolness in warm conditions and also permit sufficient breathability and permeability. Despite the growing research works in this area, there are still challenges and it is not yet been clearly realized how to incorporate all the criteria of an ideal wound dressing into one material to fulfill the multifunctional requirements in facilitating the healing procedure, cell growth, drug release behavior, and kinetics, as well as many other features. Additive manufacturing can be used to print biopolymer-incorporated cells, drugs, growth factors or even nanoparticles in the desired shape and high accuracy to be used as multifunctional-type dressings also in combination with electrospinning.

In making molecules available on the nanoscale, the maximum interaction of the drug molecules with cellular components will be obtained, which enhances the efficiency of the drugs.

Despite promising wound healing outcomes, nanofibrous wound dressings need to be still improved in various aspects. Bio-based nanofibrous dressings for wound healing are not commercially available yet, as the related industrial production is expensive and limited. This issue arises from the limitations of the electrospinning process such as low production rate, and complexity of adjusting the process on a large scale. However, several efforts are in place to produce electrospun nonwoven products using suitable multi-needle systems. Biocompatibility is also another concern to be considered owing to additives and the remaining solvent in the electrospun fibers. Specifically, biopolymers like chitosan, cellulose, and gelatin with low water solubility, are usually dissolved in toxic, acidic solvents for electrospinning [197].

In the case of active dressings, during the electrospinning process, high voltage and high shearing forces may dehydrate and harm some bioactive agents, even though collection of the mesh at room temperature often without post-treatments gives important advantages to this technology. Electrospinning is highly affected by numerous variables, which directly influence the physical– mechanical properties of the nanofibers. Hence, controlling the ultimate features of the electrospun wound dressings and release behavior of the entrapped biomolecules via those systems may be difficult and require a costly and time-consuming experimental campaign. In this regard, the electrospinning procedure must be extensively standardized, and other technologies, such as artificial intelligence could help to obtain optimally produced manufacturing [198].

Structurally, nanofibrous dressings should be engineered from the perspective of architecture, fiber arrangement and orientation, and network porosity [199]. In addition to an enhanced mechanical performance, the desired morphology and architecture of nanofibrous dressing should target the wound healing demands. Due to the various tensions applied to the dressing depending on its application place and time, the mechanical properties are very relevant. The relatively low mechanical strength, low pore size, and the difficulty in tailoring nanofibrous meshes have limited their applications as scaffolds for skin regeneration. Fabricating twisted yarns from electrospun fibers with improved mechanical properties will find advanced applications in this field. The nanofiber coating onto textile structures, in particular fabrics, filaments, and yarns, is another flexible method to provide required mechanical performance by virtue of the underlayer substrates. These proposed dressings can benefit from the characteristics of mechanical strength of the conventional spun bio- based yarns and outstanding features of nanofibers.

Stimuli-responsive fibrous materials with shape-memory properties have recently attracted great attention for their potential in regenerative medicine. Smart electrospun wound dressings combine the shape memory effect and nanofiber features with controlled delivery of biomolecules, which enhances their performance, thus appearing good candidates to fulfill the growing requirements of wound healing. Biological molecules derived from plants can be useful in protecting from oxidative stress. For example, olive leaf extract (OLE) has been revealed to protect endothelial cells (HUVECs) from ROS in a 3D model and can be useful for release from biopolymers for wound healing [200]. Further studies are aimed at incorporating OLE inside biopolymer fibers.

Application of suitable biosensors integrated into the nanofibrous dressings for monitoring wound conditions could also be another approach to develop smart materials [199]. These classes of dressings have been less developed and studied and should be further considered under the advanced wound dressing perspective. For some kinds of injuries, especially cavity wounds, the direct electrospinning of nanofibers as coating on the wound surface could also be a promising approach. Finally, great promise is held for developing novel cellulosic materials as superabsorbent for exudative wounds. Along with plant-derived cellulose, bacteria-derived cellulose shows an intrinsic hydrogel structure useful to drain serum from open wounds, thus supporting skin homeostasis and reparative processes [201]. Obtaining solvent-clean, easy, and industrially affordable methods to produce cellulose nanofibers represents the next step in this area.

## 8. Conclusions

The skin is the largest organ in the body to be damaged and any relevant wound needs prompt and effective treatment. Since wound healing is often a complex dynamic process wherein the interaction between the cells, secretory factors, and ECM molecules play an important role in the fate of the healing process, efficient wound management can result in difficulty. Biomaterial dressings should protect the wounds from environmental pollutants, provide a physiologically moist microenvironment, allow sufficient oxygen diffusion to the wound site, induce cell proliferation to facilitate tissue regeneration, while minimizing the risk of bacterial infection. The wound dressing should be soft, biocompatible, non-toxic, and non-allergenic. The healing process is greatly expected to improve by using multifunctional wound dressings. Several research works in this field have confirmed the efficiency of nanofibrous structures compared to conventional dressings. Owing to their outstanding characteristics, like small diameter, high surface to volume ratio and porous network with sufficient pore size, micro- and nano-fibers offer a great potential in the development of wound dressings. Electrospinning is a versatile and efficient method to generate fibrous structures from diverse materials with tunable compositions, features, and morphologies. Optimization of different electrospinning variables enables nanofibrous dressings to be obtained with the desired physical and biological performance. Multifunctional nanofibrous dressings possess all the requirements for effective wound healing and can be designed by blending different natural or synthetic polymers and incorporating drugs, nanoparticles, and bioactive agents through the electrospinning process. Electrospinning of natural and synthetic biopolymers from different origins, natural and renewable sources, such as cellulose, chitosan, PHAs, and PLAs have own superior features for producing dressings and tissue engineering scaffolds, thus being valuable to the current wound care industry due to their superior biocompatibility, biodegradability, bioactivity, and other specific structures. The presented review highlights the recent developments of bio-based nanofibrous wound dressings and stresses the electrospinning process as an efficient method to design an ideal wound dressing from natural and synthetic bio-based polymers in order to comply with the multifunctional requirements in facilitating the healing process, cell growth, and release behavior of drug and other bioactive agents.

## Figures and Tables

**Table 1:** Specific function and wound type targets for different electrospun biopolymer dressings.

Electrospun Mesh	Incorporated Therapeutics	Function & Wound Type	References
Cellulose acetate (CA)/Manuka honey (MH)	-	Antibacterial activity for infection in the burn wounds	[37]
CA/polyester urethane	Polyhexamethylene biguanide (PHMB)	Antimicrobial activity	[110]
Chitosan/bacterial nano cellulose	-	Antimicrobial properties	[122]
Chitosan/silk fibroin	-	Antibacterial properties Acute wounds	[121]
Chitosan/sericin	-	Biocompatibility and antibacterial properties	[120]
Chitosan/Poly (l-lactide) (PLLA)	Graphene oxide	Antimicrobial activities for chronic infected wounds	[50]
Chitosan/keratin/polycaprolactone (PCL)	Aloevera extract	Anti-inflammatory, antibacterial, antiviral, and antioxidant properties for acute and burn wounds	[35]
Chitosan/Polyvinyl alcohol (PVA)	Nanobioglass (nBG)	Biocompatibility, antibacterial activity and regeneration promotion effect for chronic wound	[5]
Gelatin/Oleoyl chitosan	-	Large skin defects or chronic wounds	[123]
Polyhydroxyalkanoate (PHA)	Dodecyltrimethylammonium chloride (DTAC) biocide	Antimicrobial effects for chronic wounds	[160]
PHA	Graphene/decorated silver nanoparticles (GAg)	Antimicrobial activity for chronic wounds	[141]
Poly (3-hydroxybutyrate-co-3-hydroxyvalerate) (PHBV)	Cerium Oxide Nanoparticle	Antioxidant and angiogenic properties Diabetic wounds	[8]
PHBV	Cerium oxide nanoparticles (nCeO2)	Antioxidant and angiogenic properties for diabetic wounds	[8]
PHBV	Curcumin	Antioxidant, anti-inflammatory, and antitumor properties chronic wounds including burns, diabetic foot ulcers, venous leg ulcers, and pressure ulcers	[39]
PHBV/cellulose	ZnO nanocrystals	Antibacterial activity for acute and infected wounds	[154]
Polylactides (PLA)	AgNPs	Antimicrobial activity for burn wounds and diabetic ulcers	[87]
PLA	Curcumin, Enrofloxacin	Antioxidant activity, antimicrobial activity, and biocompatibility	[70]
PLA	Doxycycline (DCH)	Antibacterial activity, Chronic wounds, diabetic wounds	[42]
PLA	Silver (I) metal–organic framework Ag2[HBTC][im]	Antibacterial feature	[10]
PLLA	Curcumin	Anti-inflammatory antioxidant effects	[166]
PLA/hyperbranched polyglycerol (HPG)	Curcumin	Antioxidant, anti-inflammatory and anti-infective properties for acute and chronic wound	[31]
PLA/PVA	Connective tissue growth factor (CTGF)	Diabetic wounds	[165]
poly(lactic-co-glycolic acid) (PLGA)/gelatin	Recombinant human epidermal growth factor (rhEGF), gentamicin sulfate	- Antibacterial activity and rhEGF supply - Diabetic wound	[90]
PLGA/polydopamine	Basic fibroblast growth factor (bFGF), ponericin G1	Antibacterial and cell proliferation-promoting properties for skin tissue regeneration	[89]

**Table 2:** Electrospinning-related products for wound dressing.

Product	Polymer	Device	Current Status	Case Studie or Clinical Trial Performed/Ongoing	References
Pathon	Polyurethane	Composite mesh, NO drug delivery	Clinical trial	Two double blind, randomized controlled clinical trials	[182,183]
Tecophilic™	Polyurethane-PEG	Photosensitizing-loaded mesh	Clinical trial	Comparative, 3-group based study over a total of 162 patients	[184]
SurgiCLOT^®^	Dextran	Fibrin Sealant Patch	On the market	Clinical safety and performance study in UK and Norway, Clinical safety and performance study in India, Pre-clinical comparative study of bone bleeding treated with SurgiCLOT^®^ and standard gauze, Pre-clinical Performance study of SurgiCLOT^®^ compared to Dextran-only dressing	[181,185,186,187,188]
SpinCare™	Various electrospinnable polymers	Portable electrospinning wound dressing device	On the market	Donor site wound single case Partial thickness burns single case	[189,190,191]
